# Transmissibility of severe acute respiratory syndrome coronavirus 2 among household contacts of coronavirus disease 2019‐positive patients: A community‐based study in India

**DOI:** 10.1111/irv.13196

**Published:** 2023-11-17

**Authors:** Aswathy Sreedevi, Ahmad Mohammad, Mini Satheesh, Anuja Ushakumari, Anil Kumar, Geetha Raveendran, Saritha Narayankutty, Soumya Gopakumar, Anisur Rahman, Sachin David, Minu Maria Mathew, Prem Nair

**Affiliations:** ^1^ Amrita Institute of Medical Sciences Kochi India; ^2^ WHO Country Office New Delhi India; ^3^ Government Medical College Thiruvananthapuram India; ^4^ Government Medical College Kollam India

**Keywords:** generation time, household contacts, secondary infection rate, serial interval, severe acute respiratory syndrome coronavirus 2, transmission

## Abstract

**Background:**

This study identified the risk factors for severe acute respiratory syndrome coronavirus 2 infection among household contacts of index patients and determined the incubation period (IP), serial interval, and estimates of secondary infection rate in Kerala, India.

**Methods:**

We conducted a cohort study in three districts of Kerala among the inhabitants of households of reverse transcriptase polymerase chain reaction‐positive coronavirus disease 2019 patients between January and July 2021. About 147 index patients and 362 household contacts were followed up for 28 days to determine reverse transcriptase polymerase chain reaction positivity and the presence of total antibodies against SARS‐CoV‐2 on days 1, 7, 14, and 28.

**Results:**

The mean IP, serial interval, and generation time were 1.6, 3, and 3.9 days, respectively. The secondary infection rate at 14 days was 43.0%. According to multivariable regression analysis persons who worked outside the home were protected (adjusted odds ratio [aOR], 0.45; 95% confidence interval [CI], 0.24–0.85), whereas those who had kissed the coronavirus disease 2019‐positive patients during illness were more than twice at risk of infection (aOR, 2.23; 95% CI, 1.01–5.2) than those who had not kissed the patients. Sharing a toilet with the index patient increased the risk by more than twice (aOR, 2.5; 95% CI, 1.42–4.64) than not sharing a toilet. However, the contacts who reported using masks (aOR, 2.5; 95% CI, 1.4–4.4) were at a higher risk of infection in household settings.

**Conclusions:**

Household settings have a high secondary infection rate and the changing transmissibility dynamics such as IP, serial interval should be considered in the prevention and control of SARS‐CoV‐2.

## 1 INTRODUCTION

Households provide a closed environment in which the dynamics of transmission of severe acute respiratory syndrome coronavirus 2 (SARS‐CoV‐2), such as the secondary infection rate (SIR), can be understood. Hence, the World Health Organization Unity protocol^1^ on household transmission became especially relevant, considering the increasing number of coronavirus disease 2019 (COVID‐19) cases during the peak of the pandemic, isolation of COVID‐19 patients, and the quarantine of their contacts at home. Households are ideal settings for assessing the transmissibility of pathogens and the associated determinants of susceptibility and infectivity.^2^ Social interactions within a family influence the dynamics of transmission and determine the effectiveness of mitigation strategies.^3^

A study in China estimated that the secondary attack rate (SAR) of COVID‐19 among household contacts was 12.4%, with the highest risk of infection in the oldest age group,^4^ and in Switzerland, the probability of being infected by a household member was 17.3%.^5^ Households with secondary transmission in the United States have higher living densities and median viral loads than households without secondary transmission.^6^ The SAR of household contacts ranges from 4.6% to 49.6%.^7^ This variability may also be due to the variable period of recruitment since exposure.

Various risk factors for severe COVID‐19 have been identified, such as travelling with the index patient, contact with more than one index patient, long duration of verbal contact with the index patient, spouse of the index patient, close household contact, and age > 60 years.^8^ Among close household contacts, long duration of verbal contact and sharing of a bedroom were identified as independent exposure risk factors.^8^

Symptom‐based testing strategies used for routine case detection and surveillance are unlikely to identify mildly symptomatic or asymptomatic infections that do not present in healthcare systems.^8^ More than one‐third of positive close contacts are asymptomatic, whereas symptom‐based polymerase chain reaction (PCR) testing misses more than half of infected close contacts.^7^ Therefore, accurately determining SIRs in household contacts, regardless of symptoms, remains a challenge. In the Kerala State of India, in 2021, the pandemic evolved with an exponential increase in the number of COVID‐19 cases caused by the delta variant. In March 2021, the delta variant in Kerala accounted for only 7% of total cases in the state, and it increased by more than six times to 43% in April 2021. In May 2021, 78% of the cases in the state were caused by the delta variant, which decreased to 66% in June 2021.^9^ Although the data relate to the COVID‐19 outbreak in 2021, the fact that past infectious disease epidemics and their characteristics predict the evolutionary future^10^ provides sufficient ground to document the transmissibility characteristics at various stages of the pandemic. Moreover, several viral clades cause zoonotic jumps and are likely to continue to spread within human populations. Such epidemics are often similar to previous outbreaks; for example, SARS‐CoV‐2 shares 76% of its genome with SARS‐CoV‐1.^11^

This prospective study presented an overview of the patterns of infection in household settings across three sites in southern India. This study aimed to determine the SIRs among household contacts, incubation period, serial interval, and risk factors for infection among the household contacts of the index patients in southern India.

## 2 METHODS

### 2.1 Study setting and design

This was a case‐ascertained cohort study according to the World Health Organization Unity study protocol on the Household transmission investigation protocol for COVID‐19 infection^12^ in three districts (Ernakulam, Kollam, and Thiruvananthapuram) of Kerala among the inhabitants of households of reverse transcriptase (RT)‐PCR‐positive COVID‐19 patients between January 2021 and July 2021. The three districts have the same degree of urbanisation and have tier 2 cities by the same name, which also indicates that they have low levels of pollution and moderate cost of living.^13^ They are representative of the state of Kerala and of areas across the country with a similar degree of urbanization. The recruitment occurred in a staggered manner from first quarter of 2021 in Kochi, March to April 2021 in Thiruvananthapuram, and May to June 2021 in Kollam.

### 2.2 Sampling

The sample size was determined to estimate the incubation period, serial interval, and SIR, with 95% confidence level and 7.5% absolute precision based on the studies in India and China and varied from 10 to 130.^14^, ^15^, ^16^ We considered the highest sample size of 130 for this study. Anticipating a 10% dropout rate, the sample size was calculated to be 143 patients. All eligible contacts were included in this study.

### 2.3 Operational definitions

A *household* was defined as a group of individuals sharing a common kitchen. *Index patients* in a household were defined as individuals who lived in the same household for at least 5 days prior to testing positive for COVID‐19.^17^ A list of the index patients in the study district was provided by a district surveillance team. A *household contact* was defined as any individual residing in the same household (or other closed settings) as the index patient. All identified household contacts of patients with laboratory‐confirmed COVID‐19 were included.

We followed up all contacts and index patients to determine RT‐PCR positivity on days 1, 7, 14, and 28 from the date of enrolment of the index patient (Tables S1 and S2). Serum samples to test antibody were obtained from contacts. Information on age, sex, household size, education, occupation, socioeconomic status, comorbidities, treatment‐seeking behavior, and number of household contacts, and COVID‐19 vaccination was obtained. Questions also included the date of last contact with a confirmed index patient, sharing a room, number of days the patient was ill, and information on close contacts, such as hugs, kisses, and sharing a toilet, with the index patient. We maintained a symptom diary for all contacts to record symptoms during the follow‐up.

In this study, *SIR* was defined as the frequency of new COVID‐19 infections among contacts within the follow‐up period of 28 days of incubation (range) following exposure to a primary confirmed patient in relation to the total number of exposed contacts with the denominator being restricted to susceptible contacts when these can be determined. Contacts were assumed to be susceptible if they were not vaccinated. Infection was determined by RT‐PCR positivity.^17^ The *incubation period* was calculated as the time between an exposure resulting in COVID‐19 and the appearance of the first sign or symptom of the disease in contacts of the index patient. *Serial interval* was defined as the period from the onset of symptoms in the index patient to the onset of symptoms in a contact. *Generation time* was defined as the time between infection in the index patient and infection in the household contact.^17^

*Reproduction rate* (Rt) was defined as the expected number of new infections caused by an infectious individual in a population where some individuals may no longer be susceptible^18^ and was measured approximately by dividing the number of infected individuals in contact with the number of index patients at 28 days. The socioeconomic status of the index patients was categorized according to the Government of Kerala guidelines.^19^

Nasopharyngeal swabs were collected by a laboratory technician according to the standard procedure.^20^ Swabs were collected in the household, transported through viral media in cold chain to the molecular biology lab of the base hospital and serum samples were tested for total antibodies using Wantai SARS‐CoV‐2 total IgG and IgM enzyme‐linked immunosorbent assays. The ratio of the absorbance (A) to the cutoff (C.O.) of <1 was interpreted as having no SARS‐CoV‐2 antibodies (negative) and ≥1 as having SARS‐CoV‐2 antibodies (positive). The serology of SARS‐Co‐V‐2 is not discussed in this paper.

### 2.4 Analysis

Univariate and multivariable logistic regression analyses were performed to determine the odds of infection according to household size, age, and sex. A backward stepwise logistic regression analysis was performed, considering the variables mentioned in the model. Factors associated with becoming a secondary case at 28 days, and the independent variables were occupation, comorbidities, risk of contact, severity, hospitalization, and relationship of contact with the index patient.

Adding context to the public health measures in place is the active presence of ward‐level monitoring, which ensured that the contacts of the houses stayed at home for 14 days and the index patients for 24 days or until RT‐PCR was negative. This study was approved by the ethical committee of Amrita Institute of Medical Sciences (IEC‐AIMS‐2020‐COMM‐135) on August 12, 2020. Written informed consent was obtained from the index patients and their contacts prior to enrolment.

## 3 RESULTS

**FIGURE 1:**
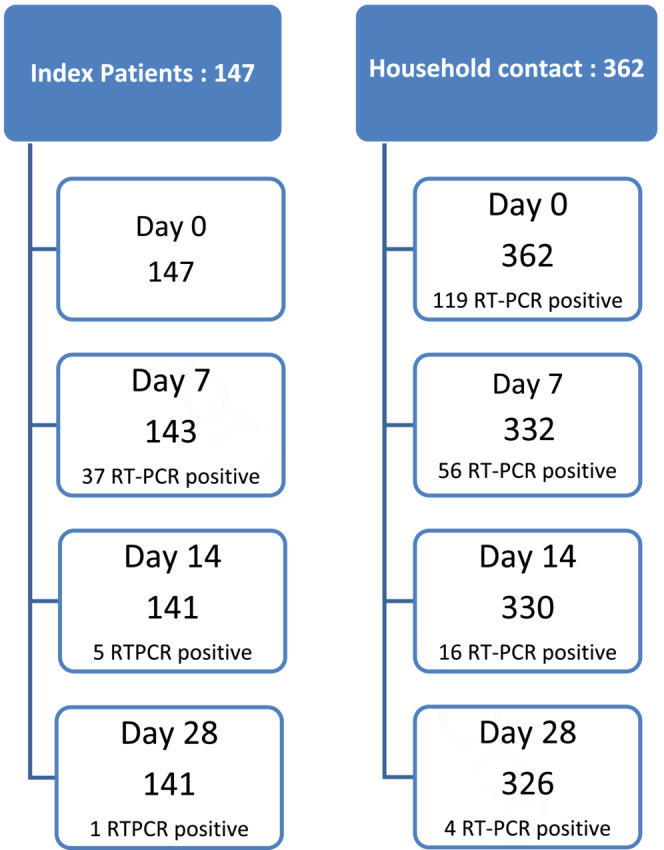
Distribution and follow up of index patients and contacts.

The recruitment and follow‐up of the patients are presented in Figure 1. In total, 147 index patients and 362 household contacts were enrolled in this study. The dropout rates were 4.0% and 9.8% among patients (141 retained) and contacts (326 retained), respectively, until day 28 from the day of enrolment.

The mean age of the index patients was 39.3 (range, 5–73) years. The mean age of the contacts was 39.4 (range, 3–94) years. Males accounted for 64.6% of the index patients and 43.7% of the contacts. The age distribution was similar among the index patients and contacts but differed significantly between sexes (*p* < 0.001). In total, 27.7% of the index patients belonged to the most economically disadvantaged group. Approximately a third of the households (31.5%) had >4 members, whereas approximately 6.9% of the households had ≤2 members (Table 1).

**TABLE 1:** Description of the study population: index patients and contacts.

Variable	Index patients (*n* = 147)	Contacts (*n* = 362)	*p* value
Age (in years)	Frequency (%)	Frequency (%)	
≤49	105 (71.4)	231 (63.8)	0.058
50–59	27 (18.36)	63 (17.4)	
≥60	15 (10.2)	68 (18.7)	
Sex			
Male	95 (64.62)	159 (43.92)	<0.001
Female	52 (35.37)	203 (56.07)	
Household size			
≤2	25 (17)	25 (6.9)	<0.001
3–4	79 (53.7)	187 (51.65)	
>4	43 (29.25)	150 (41.4)	
Severity			
Symptomatic	133 (90.4)	147 (41.4)	<0.001
Asymptomatic	14 (9.6)	208 (58.6)^†^	
^†^Contacts: seven missing data			
Socioeconomic status			
Nonpriority subsidy	53 (38.6)	Not applicable^a^	
Below poverty line	35 (25.5)		
Nonpriority	38 (27.8)		
Most economically backward	3 (2.2)		
No ration card	8 (5.8)^‡^		
^‡^Index patients: 10 missing data			
Comorbidity (≥2)			
Yes	12 (8.16)	35 (9.6)	0.595
No	135 (91.8)	327 (90.33)	

^a^ Not applicable as the contacts are from the same households as cases.

^†^ Data are not complete, and there are seven missing data in contacts.

^‡^ Data are not complete, and there are 10 missing data among index cases.

**FIGURE 2:**
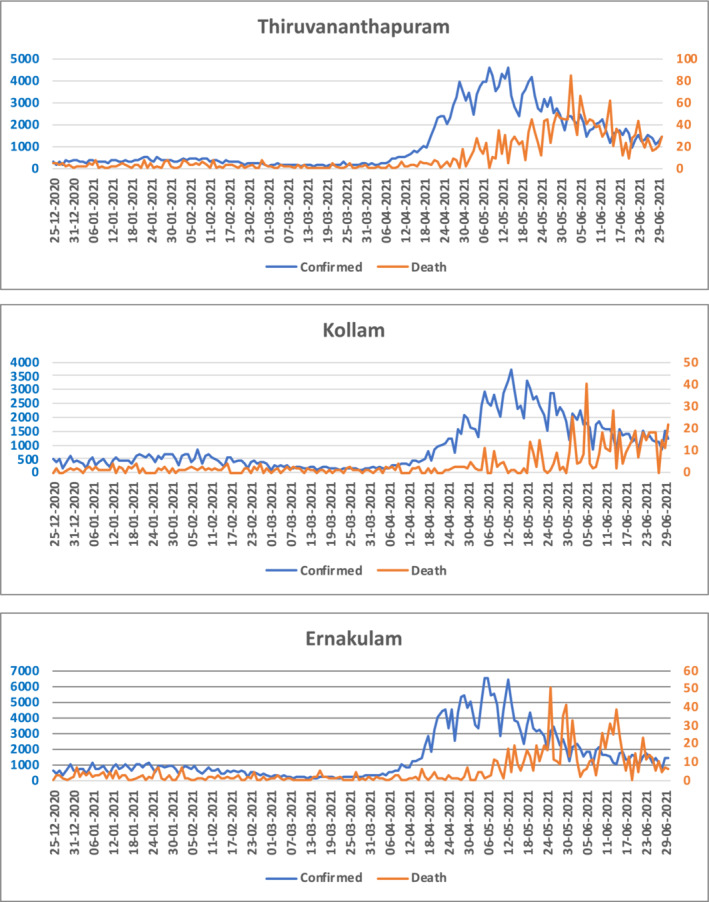
The epidemiological context of SARS‐CoV‐2 during the same period.^21^

In total, 142 (43.03%) of the 330 contacts (95% confidence interval [CI], 37.79–48.37) had positive RT‐PCR within 14 days of contact with the index patients, and 144 (44.17%) of the 326 contacts at the end of 28 days of contact with the index patients). The SIR at 28 days was 44.17% (144/326 [95% CI, 38.78–49.56]).The mean incubation period was 1.62 (95% CI, 1.3–1.94) days. The serial interval and generation time were 3 (95% CI, 2.2–3.8) days and 3.9 (95% CI, 3.6–4.0) days, respectively. Thus, the average number of individuals infected by a single COVID‐19‐positive patient (Rt) was 1.02 (144/141). Site‐wise, the Rt increased from 0.79 in Kochi to 1 in Thiruvananthapuram and 1.25 in Kollam corresponding to recruitment in the first quarter of 2021 in Kochi, March to April 2021 in Thiruvananthapuram, and May to June 2021 in Kollam. Complementary to this, the line graph of the reported confirmed cases and deaths in the three districts shows a rapid increase from April 2021, indicating the epidemiological context (Figure 2).

**FIGURE 3:**
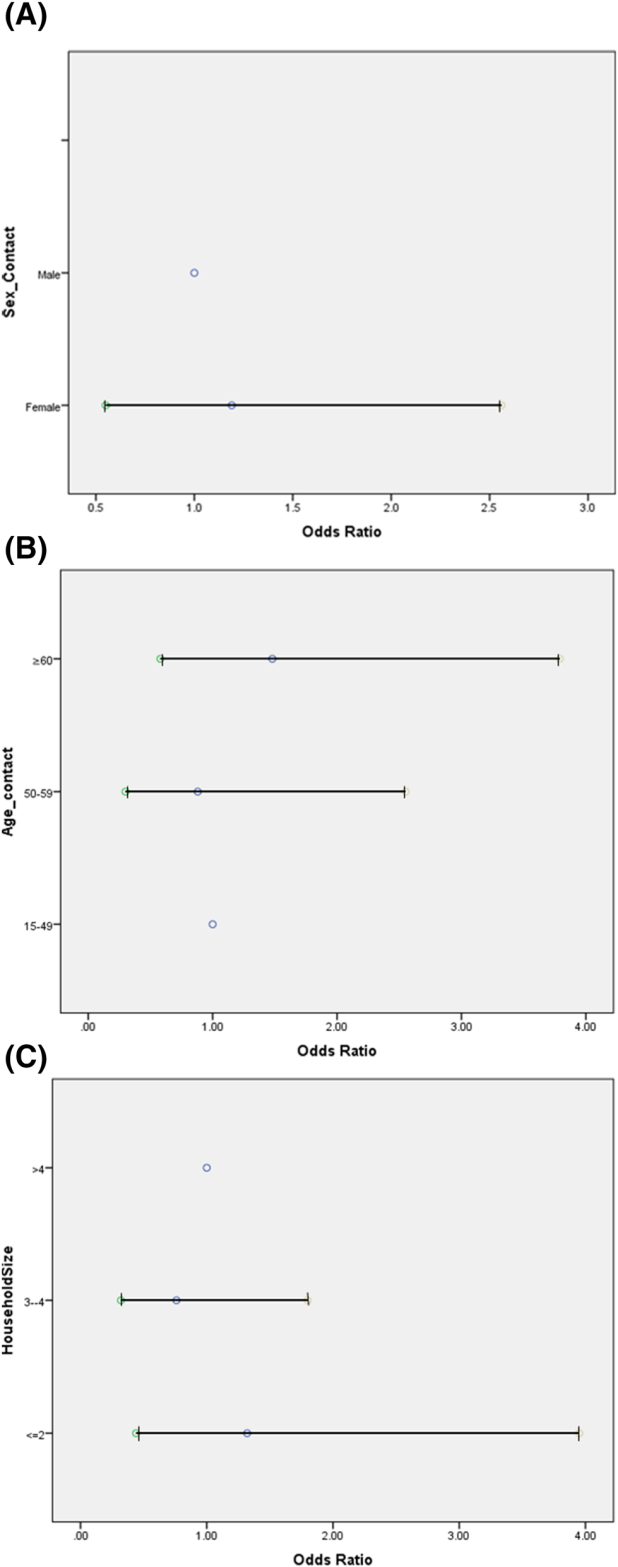
Odds of infection among contacts according to (A) sex, (B) age, and (C) household size.

The odds of becoming a secondary case were higher in a household size of 2 compared with those with a household size of >4, although the difference was not significant. In the 50–59‐year and ≥60‐year age groups, the odds of infection were 0.88 (95% CI, 0.31–2.55) and 1.49 (95% CI, 0.58–3.79), respectively (Figure 3). Although there was an increasing trend in the odds of infection with increasing age, it was not significant. The odds of infection were marginally higher among female contacts than among male contacts, although the difference was not significant (Table 2). The rate of asymptomatic cases was significantly higher in the contacts (58.6%) compared with the index patients (9.6%) (*p* < 0.001). Approximately 40.4% of the positive contacts were symptomatic. Moreover, 6.8% of the index patients and 4.3% of the contacts were vaccinated.

**TABLE 2:** Estimates of secondary infection among contacts.

Variable	Primary patients	Household contacts	Secondary patients	Secondary infection rate %	Odds of infection (95% CI)
Household size					
2	25	25	14	56	1.328 (0.446–3.950)
3–4	79	187	74	39.5	0.768 (0.327–1.804)
>4	43	150	56	37.3	1
Age of contacts					
15–49	NA	231	94	40.7	1
50–59	NA	63	21	33.3	0.883 (0.305–2.558)
≥60	NA	68	29	42.6	1.488 (0.583–3.799)
Sex of contacts					
Male	NA	159	55	34.6	1
Female	NA	203	89	43.8	1.190 (0.553–2.560)

Abbreviations: CI, confidence interval; NA, not applicable as it is the secondary infection among contacts that is being determined.

Among the index patients, RT‐PCR positivity showed a sharp decline over time by 25% in day 7, 3.5% in day 14, and 0.7% in day 28. Approximately a third (119/362 [32.8%]) of the contacts were infected with SARS‐CoV‐2 within 1 day of contact with an index patient, and 56/332 (16.8%%) were infected on day 7 of contact with an index patient, indicating a short incubation period.

Multivariate logistic regression analysis demonstrated that those who worked outside the home were protected against secondary infection (adjusted odds ratio [aOR], 0.45; 95% CI, 0.24–0.85). However, those who had kissed the index patient during illness were more than twice at risk of (aOR, 2.23; 95% CI, 1.01–5.2) developing COVID‐19 than those who had not kissed the index patient. Sharing a toilet with the index patient increased the risk by more than twice (aOR, 2.5; 95% CI, 1.42–4.64) than not sharing a toilet. However, using masks in the present setting was a risk factor for infection (aOR, 2.5; 95% CI, 1.4–4.4) (Table 3).

**TABLE 3:** Multivariate logistic regression for factors associated with secondary infection rate among contacts.

Variable	Crude OR (95% CI)	*p* value	AOR (95% CI)	*p* value
Occupation				
Worker	0.648 (0.389–1.079)	0.096	0.449 (0.236–0.854)	0.015
Student	1.055 (0.630–1.766)	0.839	0.690 (0.370–1.286)	0.242
Stay home	1		1	
Did the contact use a mask within the house when in proximity with the index patient during the time he/she was ill at home before hospitalization/being sick?				
Yes	2.058 (1.298–3.263)	0.002	2.501 (1.420–4.404)	0.001
No	1		1	
Did the contact kiss the index patient during the time he/she was ill at home?				
Yes	1.592 (0.784–3.233)	0.198	2.294 (1.004–5.242)	0.049
No	1		1	
Did the contact share a toilet with the index patient during the time he/she was ill at home before hospitalization?				
Yes	2.429 (1.538–3.834)	<0.001	2.575 (1.427–4.646)	0.002
No	1		1	

Abbreviations: AOR, adjusted odds ratio; CI, confidence interval; OR, odds ratio.

## 4 DISCUSSION

This study in southern India found a short incubation period of 1.6 days, a serial interval of 3 days, and a generation time of 3.6 days. The SIR was high at 43.0% (95% CI, 37.8–48.4), and multivariate logistic regression analysis demonstrated that the factors associated with secondary infection included working outside the home, which was found to be protective, and kissing the index patient was a risk factor for infection. Additionally, a history of sharing a toilet with the index patient significantly increased the risk of infection. The relevance of this study's findings lies in the fact that pandemics are driven by ecological, behavioral, or socioeconomic changes, zoonotic spread, and better surveillance.^22^, ^23^

The remarkably short incubation period (1.6 ± 2.6 days) in our study varies from the 6.0 days reported in studies published during this period from Guangzhou^24^ for the delta variant. However, a shorter incubation period (3.7 days) was reported in Japan with the onset of the delta variant.^25^ A short mean incubation period of 4.4 days was also reported in Guangdong during the same period as in this study.^26^ However, a systematic review reported a higher mean incubation period of 4.8–9 days.^27^ The mean incubation period in our study was shorter, possibly because of a higher number of delta variants reported.

In this study, the serial interval was 3.0 days ± 2.5. In the Guangdong Province of China, a similar serial interval of 2.3 days was reported around the same period in May to June 2021.^26^ Similarly, after the delta variant outbreak in Guangzhou, the mean serial interval decreased from 5.2 to 3.8 days.^24^ A shorter serial incubation period suggests that a substantial number of COVID‐19 cases may be transmitted in the pre‐symptomatic period. In our study, the serial interval was longer than the incubation period, possibly indicating a lack of disease transmission during the pre‐symptomatic period. The mean generation time was 2.9 days in Guangdong during the same period.^26^ We found a generation time of 3.6 ± 2.6 days.

During the initial outbreak of COVID‐19 in Wuhan, China, reproductive numbers (*R*) of 0·4 and 0.5 were observed in Shenzhen^15^ and Guangzhou, respectively.^4^ In our study, the Rt was 1.02, which could be explained by the increase in delta cases during the second quarter in the state.

In an adjoining district of Kerala, among contacts of index patients admitted to a tertiary care center in the early part of the pandemic (June to July 2020), the reproductive number was 1.2, with an SAR of 26.0%,^16^ which was higher compared with 1.02 in this study. This might be lower than that in the early part of the pandemic because only a quarter (24.5%) of the index patients were required to be hospitalized, unlike the ones in the tertiary care centre^16^ who might be sicker. Moreover, there is variability in the Rt, as measured across sites, from 0.8 to 1.25 by the end of April 2021. During this period, the Indian COVID‐19 Genome Surveillance data for Kerala showed a massive increase in COVID‐19 cases owing to the delta variant from 7% in March 2021 to 78% by May 2021.^9^

The household SIR indicates virus transmissibility,^28^ which was high in this study at 43.03%. There is evidence of the clustering of SARS‐CoV‐2 infections within households, with some households having several secondary infections, whereas others have none.^29^, ^30^, ^31^ In Shandong, China, the SAR was 39% in the first half of 2020,^32^ whereas in the United States, the SAR^6^ among household contacts was 32% in groups with high living density. The majority of cases occurred by day 7,^6^ which was also documented in this study. Reinfections seem highly improbable considering the systematic review, which indicated that protection from re‐infection remained at 78·6% at 40 weeks.^33^

The odds of infection were higher in households with a family size of 2 than in household with a family size of >4, although the difference was not statistically significant. This may be due to the nature of relationships (21.8% of the contacts were spouses, and approximately a third were children). Therefore, the relationship to the index patient was also a significant risk factor observed in studies on SARS‐CoV‐2.^34^ This may reflect intimacy, sleeping in the same room, or longer or more direct exposure to the index patients.^28^ This study also found that those who worked outside the home were protected (aOR, 0.45), whereas those who were in close proximity to the index patient, such as those who had kissed the patient during illness, were more than twice at risk of developing COVID‐19 than those who were not in close proximity with the index patient. Similarly, the contacts who had shared a toilet with the index patient (aOR, 2.5) were at increased risk.

However, the reported use of masks was a risk factor for infection (aOR, 2.5), probably due to the inappropriate use of masks, which was not examined in the present study. The various reasons may be exposure to infection before use, type of mask, and risk perception, as persons at high risk may use masks more often, whereas others might be more careless, with differential responses of those who were infected versus those who were not. In addition, due to strict legal enforcement and fear of the law regarding COVID‐19 appropriate behaviors, there may have been information bias during the interview. Biases can disrupt risk assessments in both positive and negative ways by limiting access to information, cognitive understanding, and personal experiences.^35^ Several studies on mask use have demonstrated that persistent and correct use of masks is necessary to reduce risk.^36^, ^37^ A study in Austria reported at least one erroneous behavior among approximately half (45%) of the participants, and approximately 69% touched the front of the face mask during or after adjustment.^38^ Careless handling and inappropriate use of face masks can cause self‐ and environmental contamination and increase the risk of SARS‐CoV‐2 transmission.^39^ Low adherence to use of face masks and its ineffectiveness in controlling seasonal respiratory disease has been reported.^40^ Early (within 36 h) and diligent use of masks is necessary to reduce the transmission of influenza‐like viruses.^41^ SARS‐CoV‐2 is stable on plastic for up to 72 h.^42^ Further studies are necessary to determine inappropriate mask use and the risk of infection.

Public health and social measures implemented during the pandemic, such as movement restrictions, physical and social distancing, and special protection measures for vulnerable groups may have affected the findings of this study. Regarding movement restrictions, people were encouraged to stay at home, and a policy was established to ensure that people were stepping out of their homes for necessary activities only. However, it is unlikely that this would have had a major effect on the SIR as follow‐up and assessment of the index patients and their contacts was done in their homes. Ward‐level monitoring committees ensured that the contacts of the houses stayed at home for 14 days and the index patients for 24 days or until RT‐PCR was negative.^41^ Therefore, index patients and contacts were reluctant to undergo testing on days 14 and 28, as they were apprehensive of testing positive and being subjected to further confinement at home. The potential implications of this study include understanding the transmissibility characteristics at various stages of the pandemic and its evolutionary impact.

### 4.1 Limitations of the study

Short incubation period has been estimated in this study probably due to the fact that incubation period was considered as the date of last contact with the index case and the onset of symptoms in the contact. The exposure may have occurred much before the last contact giving shorter estimates of the incubation period. We could not determine the type of masks used by the participants and some inappropriate use of masks, which may increase the risk of infection due to mask use. In addition, this study was conducted in a staggered manner across the three sites; therefore, the effects of time and variants would differ at different sites, especially the reproductive number. Severe index patients and their contacts may not have been captured in this household‐based study, because such patients required hospitalization.

## 5 CONCLUSION

Public health measures should focus on household settings and the changing dynamics of infection. Close physical contact and sharing of toilets increase the risk of infection though behavioral studies on mask use and adherence to appropriate mask use are necessary. In this study, we provide initial estimates of SARS‐CoV‐2 transmissibility in southern India, with shorter incubation period and serial interval compared with other global studies.

## AUTHOR CONTRIBUTIONS

**Aswathy Sreedevi:** Conceptualization; data curation; formal analysis; funding acquisition; investigation; methodology; project administration; resources; supervision; validation; writing—original draft; writing—review and editing. **Ahmad Mohammad:** Conceptualization; funding acquisition; methodology; project administration; resources; writing—review and editing. **Mini Satheesh:** Data curation; investigation; project administration; resources; writing—review and editing. **Anuja UshaKumari:** Data curation; investigation; methodology; project administration; supervision; writing—review and editing. **Anil Kumar:** Formal analysis; investigation; project administration; supervision; validation; writing—review and editing. **Geetha Raveendran:** Data curation; investigation; methodology; project administration; supervision; validation. **Saritha Narayankutty:** Investigation; project administration; supervision; validation. **Soumya Gopakumar:** Data curation; formal analysis; project administration; supervision; validation. **Anisur Rahman:** Data curation; funding acquisition; project administration; resources; supervision; writing—review and editing. **Sachin David:** Data curation; investigation; project administration; writing—original draft. **Minu Maria Mathew:** Data curation; formal analysis; methodology; software; supervision; validation. **Prem Nair:** Funding acquisition; project administration; supervision; writing—review and editing.

## CONFLICT OF INTEREST STATEMENT

The authors declare that there are no conflicts of interest in relation to this study.

### PEER REVIEW

The peer review history for this article is available at https://www.webofscience.com/api/gateway/wos/peer-review/10.1111/irv.13196.

## ETHICS STATEMENT

This study was approved by the ethical committee of the Amrita Institute of Medical Sciences (IEC‐AIMS‐2020‐COMM‐135 on August 12, 2020). Written informed consent was obtained from the index patients and their contacts prior to enrolment. Data from sources in the public domain have only been taken and appropriately cited.

## Supporting information


**Table S1:** Schedule of data and specimen collection in the household transmission study for Cases.
**Table S2:** Schedule of data and specimen collection in the household transmission study for Contacts.

## Data Availability

The data used in this study are available (at Amrita Institute of Medical Sciences) and can be made available after obtaining permission.
