# Supplementary material for: Transmissibility of severe acute respiratory syndrome coronavirus 2 among household contacts of coronavirus disease 2019‐positive patients: A community‐based study in India
Source: Influenza Other Respir Viruses. 2023 Nov 17;17(11):e13196. doi: 10.1111/irv.13196 (PMC10655783; doi:10.1111/irv.13196)
Supplement: Supplementary file 1 — Table S1: Schedule of data and specimen collection in the household transmission study for Cases. Table S2: Schedule of data and specimen collection in the household transmission study for Contacts. [file IRV-17-e13196-s001.docx]

Supplementary Tables

**Supplementary file Table. 1 Schedule of data and specimen collection in the household transmission**

|  | **Days Since Recruitment** | | | | | | |
| --- | --- | --- | --- | --- | --- | --- | --- |
|  | **Day 1** | **………..** | **Day 7** | **…….** | **Day 14** | **…….** | **Day 28** |
| **Home/hospital Visit and Data Collection** | **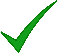** |  | **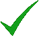** |  | **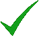** |  | **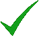** |
| **Respiratory Sample** | **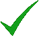** |  | **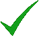** |  | **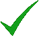** |  | **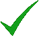** |
| **Serum Sample** | **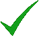** |  |  |  | **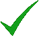** |  | **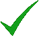** |
| **Urine & Faeces Samples** | **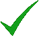** |  | **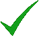** |  | **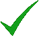** |  | **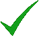** |

**study for Cases**

**Supplementary Table 2: Schedule of data and specimen collection in the household transmission study for Contacts**

|  | **Days Since Recruitment** | | | | | | |
| --- | --- | --- | --- | --- | --- | --- | --- |
|  | **Day 1** | **………..** | **Day 7** | **…….** | **Day 14** | **…….** | **Day 28** |
| **Home Visit and Data Collection** | **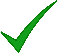** |  | **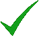** |  | **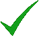** |  | **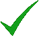** |
| **Respiratory Sample** | **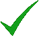** |  | **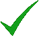** |  | **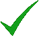** |  | **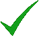** |
| **Serum Sample** | **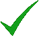** |  |  |  | **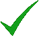** |  | **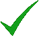** |
